# Towards a concept of embodied autonomy: In what ways can a patient’s body contribute to the autonomy of medical decisions?

**DOI:** 10.1007/s11019-023-10159-7

**Published:** 2023-06-09

**Authors:** Jonathan Lewis, Søren Holm

**Affiliations:** 1 Centre for Social Ethics and Policy, Department of Law, School of Social Sciences, The University of Manchester, Oxford Road, Manchester, M13 9PL UK; 2 Centre for Medical Ethics, University of Oslo, Oslo, Norway

**Keywords:** Authenticity, Autism, Autonomy, Body, Embodied autonomy, Medical decisions, Phenomenology, Practical identity.

## Abstract

“Bodily autonomy” has received significant attention in bioethics, medical ethics, and medical law in terms of the general inviolability of a patient’s bodily sovereignty and the rights of patients to make choices (e.g., reproductive choices) that concern their own body. However, the role of the body in terms of how it can or does contribute to a patient’s capacity for, or exercises of their autonomy in clinical decision-making situations has not been explicitly addressed. The approach to autonomy in this paper is aligned with traditional theories that conceive autonomy in terms of an individual’s capacities for, and exercises of rational reflection. However, at the same time, this paper extends these accounts by arguing that autonomy is, in part, embodied. Specifically, by drawing on phenomenological conceptions of the experience of autonomy, we argue that, in principle, the body is a necessary component of the capacity for autonomy. Secondly, through the presentation of two different cases, we highlight ways in which a patient’s body can contribute to the autonomy of treatment choices. Ultimately, we hope to encourage others to explore additional conditions under which a concept of embodied autonomy should be employed in medical decision making, how its underlying principles might be operationalised in clinical situations, and its consequences for approaches to patient autonomy in healthcare practice, policy, and law.

## Introduction

The role of the *body* has rarely been addressed in theories of autonomy.^1^ By implication, the body is distinctly absent in discussions concerning the requirements for the capacity for autonomy and the *first-person* conditions for the exercise of one’s autonomy. Indeed, apart from relational theorists taking into consideration the impact of interpersonal and social relations on autonomy (e.g., Mackenzie and Stoljar 2000; Christman 2004, 2009; Meyers 2005; Mackenzie 2008b, 2014, 2015; Westlund 2009, 2018; Anderson 2014), almost everything to do with the ways in which autonomy is specified, determined, exercised, and respected is about what goes on “in the head.” Specifically, “the head” whose relationship to the rest of the body is not explicitly considered.

For instance, the nature of autonomy is, at its most general level, usually explained in terms of the power behind the reasoning that directly gives rise to decisions, choices, or actions (Buss and Westlund 2018). Indeed, in bioethics, one’s capacity for autonomy is usually interpreted in terms of one’s cognitive capacities for reason (Schaefer et al. 2014). In addition, in medical law and medical ethics, conceptions of a patient’s exercise of their autonomy rarely extend beyond the ways in which a patient rationally reflects on, responds to, or identifies with the values, motives, and desires that underpin their healthcare decisions (Coggon and Miola 2011; Lewis 2021). At all three levels, autonomy is primarily (if not exclusively) about cognitive performance and cognitive processes.

Of course, the body and embodiment have been much discussed in the realm of phenomenology, which includes classical phenomenology (e.g., in the works of Merleau-Ponty and Heidegger, among others) and more recent phenomenological conceptions of mind, language, and action (e.g., Damasio 1994; Lakoff and Johnson 1999; Lakoff and Nunez 2000; Gallagher 2005; Johnson 2007; Clark 2008).

However, although psychiatrists and bioethicists have reinterpreted more general phenomenological ideas, with a view to, for example, developing phenomenologically oriented approaches to psychopathology and psychiatric illness (e.g., Aultman 2010; Fusar-Poli et al. 2010; Ratcliffe and Broome 2012; Catone et al. 2014; Varga 2018; Stanghellini et al. 2019; Fuchs 2020), as well as phenomenologies of illness and somatic and motor disability (e.g., Zaner 1971; Toombs 1992; Svenaeus 2000a, b; Zeiler 2010; Ratcliffe et al. 2013; Slatman 2014), there has been no explicit analysis of the role that the body can or does play in clinical decision making.^2^

Within bioethics and moral philosophy, there have been a few attempts to provide philosophical investigations into the nature of autonomy by appealing to concepts and principles in classical phenomenology (see, e.g., Mackenzie 2008a; Käll and Zeiler 2014; Hendl 2016; Slatman et al. 2016; Lewis and Holm 2022). The aims of the most developed and substantive of these accounts (i.e., Mackenzie 2008a; Käll and Zeiler 2014; Lewis and Holm 2022) have not only been to theoretically explain and justify the general idea that autonomy is embodied, affectively constituted, and—on the basis of phenomenological conceptions of selfhood—inherently relational, but also to situate phenomenological approaches to autonomy in relation to traditional, individualistic accounts and more recent—and increasingly common—theories of relational autonomy. By contrast, we do not seek to provide a full-fledged theory of embodied autonomy nor theoretically account for dimensions of autonomy that are necessarily entailed by phenomenological conceptions of experience and selfhood (e.g., the affective dimension of autonomy or its inherent relationality) (for a more holistic and theoretically rigorous overview of phenomenological autonomy, see Käll and Zeiler 2014; Lewis and Holm 2022). Rather, our focus is on some of the practical, clinical decision-making implications of the embodied experience of autonomy. Thus, in section one, we introduce the phenomenological concepts of “bodily intentionality”, “absorbed coping”, and “practical identity” to equip us with the most essential tools to investigate some of the contextual and bodily factors that influence a patient’s ability to exercise their autonomy in a clinical encounter and thereby understand some of the medical decision-making implications of an embodied approach to patient autonomy. Subsequently, by further clarifying the nature and implications of the phenomenological concepts of “bodily intentionality”, “absorbed coping”, and “practical identity” through discussions of two pseudo-anonymised, part-fictional clinical cases, section two argues that the autonomy of a patient’s treatment choice can sometimes substantively depend on their bodily capacities and expressions. Importantly, previous accounts of embodied autonomy have justifiably tended to emphasise the autonomy-constituting role of the lived body, of which we attempt to provide an overview in section one, as well as more abstract, phenomenological explanations for why autonomous decision-making rests on pre-reflective bodily autonomy (see Käll and Zeiler 2014; Lewis and Holm 2022). By contrast, an important part of this paper involves an analysis of how a patient’s autonomy relates to their experience of “bodily disunity”, i.e., a loss of bodily control and/or a feeling of alienation from one’s body that coincides with one’s inability to practically engage with one’s environment in the ways one is disposed to (Meyers 2005, 39; Kong 2017, 78). Specifically, we seek to explain the ways in which experiences of bodily disunity relate to and influence a patient’s mental capacity and cognitive processes when attempting to exercise their autonomy.

## The body as a component of autonomy

Before arguing that the body, or, more specifically, the patient’s body, contributes to their autonomy, it is important that we make two clarificatory points. Firstly, our discussions of “the body” should always be understood as referring to an individual’s body with its specific configurations and activities, and its specific history of lived experience—“my body” as a patient would say—rather than in some generic way as relating to “a body” or “the body.”

Secondly, when we claim that, in principle, the body is a component of a patient’s autonomy, we do not mean that the body, in and of itself (i.e., independently of one’s cognitive capacities for reason), is a sufficient condition for the capacity for, and exercise of autonomy. Indeed, even for phenomenological conceptions of autonomy, the capacity for reason is a necessary condition for one’s capacity for, and exercise of one’s authority over one’s actions (Crowell 2013; Wrathall 2015).

By “capacity for reason,” medical law typically interprets this concept in terms of competence. In the United Kingdom, for instance, competence is explicated in Sect. 3(1) of the Mental Capacity Act 2005 as the capacities to understand, retain, use, and weigh information relevant to a decision and communicate that decision. Similarly, as G. Owen Schaefer, Guy Kahane, and Julian Savulescu (2014, 126) argue, when it comes to medical ethics, competence conditions tend to refer to “reasoning capacity”, specifically, the cognitive capacities needed for an individual “to properly comprehend the options ahead of them, evaluate different options, deduce appropriate courses of action, weigh consequences, etc.” These approaches are broadly line in with certain phenomenological interpretations of the capacity for reason to the extent that, according to the phenomenologist Steven Crowell, such interpretations understand it as the capacity for “articulat[ing] *courses of action*—weighing evidence and considering reasons for going on in one way or another” (Crowell 2013, 202).

Thus, even for phenomenological approaches to autonomy, the capacity for autonomy is still partly determined by first-person, cognitive conditions linked to one’s capacity for reason. Furthermore, when it comes to the exercise of autonomy, standard approaches in bioethics, medical law, and classical phenomenology are united to the extent that they recognise that whether one exercises one’s autonomy or not depends, in part, on those motivating attitudes and experiences in which one discovers oneself to be an ineliminable ground of one’s decisions and actions. Thus, the exercise of autonomy, even of a phenomenological kind, is constituted by cognitive processes of rational self-reflection.

However, for phenomenologists, there is also an important non-cognitive dimension to autonomy that, as a matter of principle, cannot be accounted for by appealing to the capacity for, and process of cognitive reflective self-awareness.

Key to understanding this non-cognitive dimension is the phenomenological principle that one’s *primary* intentional and meaningful access to the world is always through, and in relation to one’s body (Merleau-Ponty 2002, 139 − 40). According to phenomenologist Thomas Sheehan, our primary experiences of and in the world are intentional in the sense that the bodily movements and embodied perceptions that constitute such experiences are always *directed at* or *about* something that is a correlate of a human practice (Sheehan 2014,128). Furthermore, these non-cognitive experiences are meaningful in the sense they always involve interpretation, that is, “things out there in the universe come to be seen as meaningfully present phenomena: the perceived of a perception, the loved of an act of love, the judged of an act of judgment” (ibid.). Importantly, these intentional bodily interpretations of phenomena cannot be reduced to acts of cognition (Husserl 1982; Heidegger 1995; Merleau-Ponty 2002; Smith 2005; Sheehan 2014). In other words, we interpret and encounter phenomena as *inherently and immediately* meaningful before we turn them into objects of reflection (Merleau-Ponty 2002, 162). This idea that an individual is intentionally involved with their environment through bodily movements and practical actions that cannot be equated with a cognitive outcome (e.g., deliberation on their desires and beliefs) has been referred to as “bodily,” “operational”, “practical,” and “non-propositional intentionality” (Dreyfus 2000, 2014; Wrathall 2011; Gallagher 2012; Crowell 2013; Käll and Zeiler 2014).

What does the phenomenological principle of non-cognitive intentional and meaningful experience mean for autonomy? Firstly, it means that even before one begins the process of reflecting on one’s values, desires, and motivations as part of one’s exercises of one’s autonomy, one has already interpreted one’s values *as* values (i.e., in the sense that they fall under the concept “value”). Secondly, my cognitive access to my values, desires, and motivations is dependent on *contextualised*, practical bodily engagements, that is, bodily comportments and practical actions that are always part of a context which I identify as significant and meaningful for me (Smith 2005; Crowell 2013; Sheehan 2014; Dreyfus 2014). Thus, from a phenomenological perspective, one’s primary non-cognitive, practical engagements not only disclose values *as* values, but also interpret values *as* specific values, *as* one’s values, and *as* values that make sense in terms of the other attitudes and commitments that one holds as well as in terms of one’s lived experiences (Lewis and Holm 2022).

Key to understanding why, for phenomenologists, practical bodily engagements are the primary means of accessing one’s *authentic* motivating attitudes (i.e., in the sense that they are of personal significance, and thereby interpreted as being one’s *own* reasons for acting) are the related concepts of “absorbed coping” and “practical identity.” In the rest of this section, we will articulate these two phenomenological concepts and outline how they explain one’s ability to disclose one’s motivating attitudes.

Hubert Dreyfus, a phenomenologist and classical phenomenology scholar, endorses the concept of “bodily intentionality” (i.e., that individuals are intentionally involved with and in the world through active bodily engagements that cannot be conflated with the deliberative outcome of their values, desires, and motivations). However, Dreyfus extends the concept in one important sense: intentional bodily engagements through which one interprets phenomena as inherently and immediately meaningful are to be understood as “a steady flow of skilful activity in response to one’s sense of the environment” (Dreyfus 2014, 81). In other words, “one’s body is solicited by the situation to get into the right relation to it,” “something like what athletes call flow, or playing out of their heads” (ibid.). What results is a state of “bodily unity,” that is, a state of “equilibrium” or “poise” that exists between one’s body and one’s bodily, practical engagements with one’s environment (Kong 2017, 78–81). The idea that intentional bodily engagements are skilful, flow-like, unifying actions unmediated by cognitive states or processes is referred to as “absorbed coping” (Dreyfus 2000, 2014) or “engaged coping” (Crowell 2013, for discussions of the same phenomenological principle, see also Meyers 2005; Wrathall 2015). Obvious, publicly recognisable examples of absorbed coping would include the performances and practices of professional musicians, dancers, carpenters, surgeons, athletes, chefs, and so on. Although individuals who initially take up these activities or who are planning a new activity will need to cognitively engage with specific tasks and tools, the point is that once they have mastered them and when engaged in the practice, rational analysis or a cognitive “stepping-back” from these tasks is, in general, no longer necessary—indeed, according to Dreyfus, skilful and masterly engagement in these practices is (ironically) conditional on one’s cognitive unawareness of one’s skills (Dreyfus 2014, 95 ff., also see interviews with professional jazz musicians, carpenters, chefs, dancers, and flamenco artists in Ruspoli 2010). When absorbed in a practice, masters of their respective trades are, as Dreyfus (2014, 81) suggests, in a state of bodily “flow”, a “playing out of their heads”. But instances of absorbed coping can also be much more mundane (see, e.g., Meyers 2005). In short, the concept of absorbed coping captures all those contextualised bodily mannerisms, comportments, practices, and routines that we express, carry out, and are disposed to but of which we only become cognitively aware when we stop to think about what we’re doing or when we discover that we can no longer do what we’re disposed to do (Crowell 2013, 249).

As these examples imply, how we express absorbed coping through our intentional bodily actions is specific to each of us. According to Dreyfus, one’s fluid, skilful actions are always “in response to *one’s sense* of the environment” [emphasis added] (Dreyfus 2014, 81). This leads us to the second key concept that underpins the more general phenomenological concept of bodily intentionality. Specifically, what grounds “one’s sense of the environment” and, simultaneously, disposes one’s body to intentionally respond to that environment in particular ways is one’s “practical identity” (Crowell 2013; Wrathall 2015). According to Crowell (2013, 218), one’s practical identity describes the “for-sake-of-which” I do or do not do something (e.g., making a birdhouse for the sake of *being* a carpenter or not isolating myself from social interactions and situations for the sake of *being* an extrovert). Such descriptions include doctor, mother, lover, liberal, runner, cyclist, cynic, autistic, and so on. As Crowell acknowledges, most people’s practical identity is an evolving, complex constellation of many descriptions. Furthermore, such identities are practical to the extent that “they are not primarily objects for theoretical contemplation, nor merely social roles that are attributed to us in a third-person way, but are expressed in what we do” (ibid., 243). Indeed, descriptions will be associated with some “constitutive standards” for practical action by which one can assess oneself or be assessed by others as succeeding or failing to embody a particular description (ibid.). But even constitutive practical actions (i.e., those that one would be expected to perform on the basis that one identifies with a particular description) will be expressed by individuals in specific ways. Moreover, given that an individual inhabits many descriptions, some will be valued more than others and thereby feature more prominently in an individual’s intentional bodily engagements, meaning that everyone will have their own unique practical identity, expressed in specific ways.

Returning to the question of why, for phenomenologists, one’s practical, cognitively unmediated bodily responses to one’s sense of the environment interpret values, desires, and motivations as being authentic, the point is that one’s skilful, fluid, and intentional actions (i.e., absorbed coping) are expressions of one’s practical identity. Through embodied expressions of my practical identity, I am accessing and disclosing my own values, desires, and motivations because my practical identity already provides me with these reasons for acting in certain ways (Crowell 2013, 243). As Crowell explains, “I hammer nails in order to secure boards, but such action has a self-referential dimension as well: I am *trying* to be a carpenter; being one (practically) is an *issue* for me, is at stake in what I do” (ibid., 244-5). Furthermore, because we embody a specific practical identity with its own skilful, fluid bodily expressions, the state of bodily unity that we achieve through absorbed coping will be specific to each of us. In other words, we achieve practical “equilibrium” or “poise” in our environments through our own bodily mannerisms, comportments, practices, and routines.

Having outlined the concepts and central principles of bodily intentionality, absorbed coping, and practical identity, and the ways they relate to one another, we can infer from these discussions three phenomenological implications of pre-reflective, intentional bodily action for the concept of autonomy.

Firstly, as explained, the ability to experience one’s values as inherently and immediately meaningful, that is, to interpret values *as* values, *as* one’s values, and *as* values that make sense in terms of the other attitudes and commitments that one holds, is dependent on having a practical identity, which is expressed through intentional bodily engagements with one’s environment. As a matter of phenomenological principle, individuals without a practical identity (i.e., descriptions under which they value themselves) would have no reasons for acting and, relatedly, no values, desires, or motivations on which to reflect when exercising their autonomy in decision-making situations (Crowell 2013, 243−50). Thus, they would be incapable of intentionally and meaningfully engaging with their environment through practical bodily actions. Moreover, given that intentional bodily engagement is, as a matter of phenomenological principle, a necessary condition for explicit deliberation or rational reflection on one’s values, they would be incapable of exercising their autonomy (ibid.). Thus, having a practical identity and thereby the capacity for bodily intentionality is a necessary component of one’s capacity for autonomy.

Secondly, one skilfully, intelligently, and corporeally navigates and responds to one’s environment in accordance with one’s practical identity, which provides reasons for acting in certain ways. When I express my practical identity through bodily engagements and thereby achieve a state of bodily unity, I am, by implication, pre-reflectively accessing and enacting my authentic traits, values, desires, and motivations. In other words, through absorbed coping, I am pre-reflectively validating or disowning attitudes that constitute my reasons for action (Meyers 2005, 45–48). To the extent that one accepts this phenomenological principle, it follows that our intentional bodily engagements can be viewed as a way of non-cognitively exercising our autonomy (see also, Käll and Zeiler 2014).

The third implication of bodily intentionality for autonomy concerns the ways in which pre-reflective bodily experience of one’s motivating attitudes relate to decision-making situations, that is, those situations where one cannot just rely on one’s bodily actions to (non-cognitively) exercise one’s autonomy, but where we’re required to reflect on our reasons for action and communicate an explicit choice. This question raises complex issues, requiring detailed analysis as well as illustrative examples, so we afford it its own section.

## What does the body mean for autonomy in medical decision making?

According to the phenomenological principles of bodily intentionality and absorbed coping, our authentic motivating attitudes are disclosed to us and enacted even before we come to reflect on or rationally respond to them. But what happens when we are required, as patients, to consent to or refuse a particular medical intervention or choose from a range of treatment options? It is nonsensical to suppose that our bodily engagements are a sufficient condition for exercising our autonomy in these contexts because, firstly, such enactments of our values and desires are—despite being the basis of our cognitive reflections—pre-reflective and non-cognitive. In addition, making medical decisions in relation to a specific condition will, for most of us, not be part of our everyday practices (i.e., being a patient will, for most people, not be something they conceive as part of their practical identity). When we are required to take responsibility for ourselves (e.g., when making medical decisions that concern us), we experience a cognitive disengagement from our everyday fluid, skilful practices of absorbed coping and are solicited by the situation to choose to make a choice (for details, see, e.g., Braver 2014; Crowell 2013; Wrathall 2015). Therefore, reason and reasoning are necessary conditions of autonomy; in order to exercise our autonomy in decision-making contexts, we are required to rationally reflect on or respond to our own values, desires, and motivations. However, according to phenomenological principles, there is not a sharp break between the pre-reflective self-awareness constitutive of one’s bodily intentionality and one’s cognitive, rational reflections on one’s values, motives, and desires. The point is that one’s motivating attitudes are always already cognitively available to us because of the practical identity we embody. In addition, one’s pre-reflective self-awareness remains in view when we come to reflect on how we act or which choice to make.^3^ In other words, detached reflective self-awareness, which is traditionally the hallmark of autonomous decision-making, is only possible because there is a prior pre-reflective self-awareness built into experience. This means that, in decision-making contexts, a patient can reflect on those values that they initially disclosed at a pre-reflective level and thereby employ them as reasons for their decision (for a more detailed explanation of the link between bodily intentionality and autonomous decision-making, see Käll and Zeiler 2014; Lewis and Holm 2022).

Because my practical identity is the source of my values and desires on which I base my decisions and choices, and because my practical identity is something that, by definition, “expresses ‘me’” (Crowell 2013, 243), some who have written on the phenomenology of illness have argued that when we make medical decisions we do so with the aim of preserving or—if a particular condition has temporarily impaired our ability for absorbed coping—re-establishing our practical identity (Svenaeus 2000a, b; Meyers 2005; Kong 2017). In other words, we are disposed to choose the treatment that will best allow us to go back to the way we practically comported ourselves in everyday life before the onset of illness (i.e., to re-establish our practical identity and a state of bodily equilibrium or poise in our everyday environments). However, for most patients, it is likely that their treatment deliberations would involve no substantive consideration of their practical identity, absorbed coping, or their body in general.

Take, for example, an otherwise healthy, young adult patient with no underlying conditions or impairments who goes to their GP seeking treatment for what turns out to be a minor, early-stage staph infection on a small part of their torso, and who, subsequently, agrees to the GP’s recommendation of a short course of antibiotics. It would seem entirely reasonable to accept that the patient has the capacity for reason and has exercised that capacity in making a treatment decision in accordance with their underlying values. Even though such an assumption is perfectly compatible with an embodied approach to autonomy (given that the capacity for and exercise of reason are necessary conditions of phenomenological conceptions of autonomy), it implies that, in most instances, there is no apparent reason to look beyond traditional cognitive approaches to autonomy prevalent in the medical ethics literature.

Assuming that, for most people with relatively minor and easily treatable medical conditions, exercising autonomy in treatment contexts will primarily depend on their rational reflections on their motivating attitudes, the discussions in section one raise theoretically interesting and, at least in bioethics, previously unexplored ideas and claims about the source of our authentic reasons for action. However, even though having a practical identity and thereby the capacity for bodily intentionality is, in principle, a necessary component of one’s capacity for autonomy, it is not yet clear why the body can play a substantive role in medical decision making.

As shall be explained through a phenomenologically oriented analysis of two clinical cases, two conditions under which the body can contribute substantively to the autonomy of medical decisions are (i) when a patient experiences bodily disunity and expresses themselves in ways which a clinician judges to be indicative of irrationality and/or a lack of autonomy; and (ii) when a patient experiences bodily disunity as a result of the condition with which they presenting and will never again be able to embody the practical identity they had before the onset of that condition. The common factor in these cases is the patient’s experience of “bodily disunity”. As explained above, when one is skilfully engaged in a practice in a way that is unmediated by cognitive states and processes (i.e., absorbed coping), one achieves a state of “equilibrium” or “poise” with one’s body and one’s bodily engagements with one’s environment. This state of “bodily unity” is, from a phenomenological perspective, considered to be normatively significant for two reasons; firstly, according to Kong (2017, 80), we experience “a sense of satisfaction when we manage to achieve it”; secondly, as detailed previously, it allows us to pre-reflectively access our authentic values, desires, and motivations, which we can then employ as reasons that underpin the rationality of our decisions and choices. By contrast, bodily disunity refers to loss of bodily control that coincides with a loss of practical “equilibrium”, i.e., an inability to practically engage with one’s environment in the ways one is disposed to (Meyers 2005, 39; Kong 2017, 78). Again, the experience of bodily disunity is normatively significant in the sense that, firstly, one can find the alienation from one’s body “profoundly disorienting” (Meyers 2005, 39, also see Svenaeus 2000a, b). Secondly, it can either, as the first case illustrates, temporarily inhibit the patient from cognitively reflecting on treatment options or cognitively accessing their underlying values, desires, and motivations, or, as shall be shown in the second case, it can lead to a crisis in one’s practical identity such that the patient’s reasons for action can no longer be rationally employed to underpin a genuinely autonomous treatment decision. In the first case below, the patient’s experience of bodily disunity stems from a combination of their autism and situational factors within the clinical encounter. In the second case, the source of bodily disunity is an uncurable (somatic) condition. However, experiences of bodily disunity can also result from pain, injury, illness, impairment, or features of the clinical decision-making situation (e.g., whether it is an emergency, whether it is stress-inducing for the patient, whether the clinician conducts themselves in a professional, empathetic, compassionate way), or a combination of these factors. Although the sources of bodily disunity in the following two cases are different, what unites them is that the patients’ respective experiences of bodily disunity inhibit their autonomy. In addition, as shall be explained in what follows, purely cognitive approaches to autonomy cannot *adequately* explain why each patient is unable to exercise their autonomy nor *adequately* account for those conditions that need to be satisfied so they can genuinely exercise their autonomy in these specific medical decision-making contexts. Moreover, to avoid unwarranted and unnecessary paternalism or substituted decision making and, as relational theorists of autonomy have convincingly argued (e.g., Mackenzie 2008b; Dodds 2014; Mackenzie et al. 2014), prioritise obligations to support patients in exercising their autonomy, healthcare mechanisms for facilitating patient autonomy in these instances would require knowledge and consideration of the embodied dimension of patient autonomy. In other words, to support patients to exercise their autonomy in a specific decision-making context, the support offered by healthcare professionals would need to be targeted at the patient’s body rather than at their psychological states and processes.

### Case 1: Stimming in patients with autism spectrum disorder

*Seb is a 22-year-old with autism. Nevertheless, he is high-functioning, generally competent, and demonstrably able (under the right conditions) to understand, retain, use, and weigh medical information and communicate treatment decisions. Seb has no underlying health conditions or any other cognitive or somatic impairments. Seb goes to his GP seeking treatment for what turns out to be a minor, early-stage staph infection on a small part of his torso. The waiting room is noisy and busy. Given the sheer volume of patients and the complexity of some of the cases, Seb’s GP is running 45-minutes late. Seb’s case is comparatively straightforward, so, when he enters the treatment room, the GP quickly diagnoses the issue, rapidly informs Seb about the diagnosis and his treatment options, and recommends a short course of antibiotics. While informing Seb about the condition and the associated treatment recommendation, the GP notices that Seb is repetitively and rhythmically “flapping” one of his arms. Conscious of the number of patients he has to attend to, the GP presses Seb for an answer about whether he agrees to the treatment recommendation. Continuing to “flap” one of his arms, Seb begins to make repetitive, rhythmic “clicking” noises with his tongue. The GP, once again, presses Seb for an answer. Seb’s movements become more emphatic and his vocalisations louder. The GP asks Seb whether everything is okay. Seb says “yes,” but still does not provide an answer to the GP’s treatment recommendation. The GP tells Seb that he will call Seb’s mother and discuss the diagnosis and treatment recommendation with her. He instructs Seb to return to the waiting room. Overwhelmed by the noisy and busy waiting room environment, Seb leaves the surgery.*

Before proceeding with a phenomenological analysis of this case, it is worth pointing out that the following discussions will not be directly concerned with the GP’s actions. We have discussed some of these issues elsewhere (Lewis and Holm 2022). Rather, our primary aim is to focus on Seb’s active bodily engagements in this clinical environment and interpret them in relation to his temporary inability to exercise his autonomy. Secondly, we assume that Seb is capacitous for the purposes of mental capacity law. Although we believe that the principles of embodied autonomy have specific implications for how non-capacitous patients should be treated in clinical decision-making contexts, these implications lie beyond the scope of the current paper.

The first thing to draw attention to, at least where a patient’s experience of their own body in this situation is concerned, is that upon entering the treatment room, and increasingly throughout the consultation, Seb exhibits what—to this part-fictional GP—appears to be heteronomous and irrational movements, which, when considered alongside a patient’s seemingly irrational decisions or inability to decide, are often taken to be indicative of an absence of autonomy (Faso et al. 2015; Nolan and McBride 2015; Parsi and Elster 2015; Sheppard et al. 2016; Graber 2017; Kapp et al. 2019; Späth and Jongsma 2020).

Autistic adults have reported exhibiting repetitive, usually rhythmic bodily movements and vocalisations—“self-stimulatory behaviour” or “stimming” (Nolan and McBride 2015)—in response to distorted or overstimulating perceptions and dysregulated, excessive, or distracting thoughts, all of which are very often triggered by confusing, unpredictable, or overwhelming environments (Kapp et al. 2019, 1786). Individuals with autism “stim” because they generally find it more difficult to govern their thoughts and actions in such environments due to—depending on the theory one holds—excessive, hypersensitive, insufficient, or inefficient sensory processing (Bertone et al. 2005; Miller et al. 2007; Baron-Cohen et al. 2009; Haigh 2018; Kapp et al. 2019; for an alternative account, see Pellicano and Burr 2012).

Moreover, according to Kong (2017, 51–99), reports by autistic individuals concerning their experiences of stimming suggest that such experiences can be captured by the concept of bodily disunity in the sense that stimming indicates an absence of practical unity, equilibrium, or poise in their environment. As Williams (1999, 43) reports first hand, sensory overload “makes the body react as if being attacked or bombarded.” Affirming and elaborating on this point, an autistic adult describes the experience of being overwhelmed by sensory information as undermining his usual practical engagements with his environment: “I began to fear all those unknown paths, clothes, shoes, chairs and strange human voices. Each one challenged me by putting me in front of a new situation for me to face and understand…” (Bogdashina 2005, 60).

However, stimming is not just indicative of a temporary lack of experienced bodily unity. According to autistic adults, it also functions as a means of coping with uncertain and overstimulating environments, and thereby affording them a level of self-control over their bodies, their affective states, and the cognitive processes that have traditionally been theorised as the primary components of autonomous choice (Davidson 2010; Kapp et al. 2019). These claims have also received theoretical support (see, e.g., Pellicano and Burr 2012).

When interpreted as a corporeal means of better coping with those environments that give rise to an experience of bodily disunity, stimming should not be perceived as an irrational or involuntary response indicative of a lack of autonomy. This coincides with the broader phenomenological principle that active bodily engagements are not irrational in the sense that they can be contrasted with rational, cognitive acts (Lewis and Holm 2022). Rather, as previously explained, bodily expressions are a necessary condition of cognitive self-awareness, and, therefore, they are much more “rational” than usually conceived outside of the phenomenological tradition.

Although his affective and bodily distress renders Seb unable to immediately exercise his autonomy in response to the GP’s questions, the point is that his repetitive, rhythmic motor movements and vocalisations are helping him re-establish a sense of bodily unity in accordance with his practical identity, which, in turn, as autistic adults have acknowledged and as a phenomenological approach to autonomy entails, makes detached reflective self-awareness possible. Viewed in this way, stimming serves to place an individual with autism in a position to exercise their cognitive capacities and thereby exercise their autonomy. In Seb’s case, had the environment within the GP’s waiting room been less overwhelming, had there not been such a long waiting time, had the GP not rushed the consultation and continued to press Seb for a response to the treatment question, or had the GP understood the relationship between Seb’s stimming and his inability to make an immediate treatment decision, then, in principle, Seb would have been in a better position to cognitively reflect on the GP’s recommendation in light of his own motivating attitudes and thereby make an autonomous choice.

Not only does the analysis of this case serve to underscore the theoretical discussions in section one, specifically, that expressions of one’s practical identity and achievement of a state of bodily unity are what makes cognitive engagement with one’s authentic motivating attitudes possible, but also it suggests that, in those cases where are patient is exhibiting seemingly irrational behaviours during the medical decision-making encounter, healthcare practitioners should consider the possibility that such expressions are not irrational per se and that the patient’s seemingly irrational decisions or inability to choose could be principally influenced by their bodily experiences. Indeed, although this case focuses on a patient with autism, the concepts and implications discussed would, in principle, apply to any patient who is displaying seemingly irrational behaviour because of an experience of bodily disunity, which temporarily inhibits them from cognitively reflecting on treatment options. This could, for example, include patients experiencing stress, anxiety, uncertainty, fatigue, or other forms of bodily or affective distress as a result of their present condition or the clinical encounter (for a discussion of these more common and straightforward clinical cases, see Lewis and Holm 2022). According to the phenomenological principles outlined in section one, if such patients have mental capacity and are supported to establish a state of practical bodily equilibrium or poise in the clinical environment, then they would be able to cognitively access and rationally reflect on or respond to their values, desires, and motivations and thereby exercise their autonomy accordingly. Of course, we recognise that in certain medical situations (e.g., emergencies), it may not be possible to provide such support in a timely fashion without the patient experiencing serious long-term or fatal health consequences. In general, the preceding phenomenological analysis would be expected to apply to medical conditions for which a potentially extended or delayed period of decision making would not engender serious health or well-being consequences.

Importantly, purely cognitive conceptions of autonomy can neither fully explain why patients in such circumstances are unable to exercise their autonomy nor offer a principled basis for developing mechanisms to support their autonomy in the clinical encounter. Although it is true that in these contexts patients are temporarily unable to cognitively access and rationally reflect on or respond to values, desires, and motivations, identifying autonomy purely with cognitive capacities and processes has problematic implications. It can lead to the assumption that the source of autonomy inhibition is to be found in the patient’s cognitive capacities for autonomy (i.e., their competence), indicating some form of cognitive impairment, or in their failure to exercise those capacities for some unrelated (epistemic) reason (e.g., a failure to comprehend information provided by the clinician or a failure to understand that they are required to make a decision). When considered in relation to a framework that identifies autonomy purely with capacities for reason and cognitive processes of rational reflection, seemingly irrational or socially unacceptable behaviours could be judged as offering support to this assumption and, as a matter of principle, lead one to infer that the patient, rather than being temporarily inhibited from making an autonomous decision, is cognitively incapable of making such a decision altogether. For instance, although some individuals with autism can be cognitively impaired (Matson and Shoemaker 2009; Goldin et al. 2014), a diagnosis of autism is taken as a reason to be sceptical about autonomy in general (Parsi and Elster 2015; Graber 2017; Späth and Jongsma 2020). Indeed, the conflation of autonomy, cognitive capacities, and rational behaviour has led to assumptions that autistic individuals are not self-aware or able to develop or organise a way of life according to their preferences, goals, and interests (Späth and Jongsma 2020). Furthermore, purely cognitive conceptions of autonomy, through which stimming would be viewed as an expression of irrationality, lend support to interventions to eliminate, modify, or reduce stimming (Lanovaz et al. 2013). This would not only, in principle, disrupt autistic people’s capacity for, and exercises of autonomy, but also, as some have argued, undermine their well-being (Robeyns 2016; Rodogono et al. 2016), and violate principles of medical ethics (Nicolaidis 2012). These negative and often misleading descriptions of people with autism have been challenged by recent evidence on the lived experiences of neurodivergent individuals and positions within the neurodiversity movement (see, e.g., Kapp 2019; Humpston and Broome 2020; Milton et al. 2020; Pellicano and den Houting 2022; Rice-Adams 2023). Not only do they call into question attempts to eliminate motor stereotypies, which remain popular both clinically and in research, but they also suggest that clinicians should pay greater attention to the lived experiences of neurodivergent patients in order to support their involvement in decision-making and facilitate interventions that allow them to lead fulfilling and autonomous lives *with* their symptoms.

Whereas this case illustrates the body’s substantive contributions to the autonomy of medical decisions when a patient expresses themselves in ways which a clinician judges to be indicative of irrationality and/or a lack of autonomy, the following case focuses on the role the body performs when a patient experiences bodily disunity as a result of the condition with which they presenting and will never again be able to embody the practical identity they had before the onset of that condition.

### Case 2: The autonomy-undermining effects of fibromyalgia

*Mairéad is forty years’ old. Outside of her career as a mechanical engineer, she is a long-standing member of numerous amateur sporting societies for tennis, cross-country running, road cycling, and bouldering. All her friends belong to one or more of these societies and all her meaningful social interactions take place within these recreational contexts. She describes sport as her “way of life”. For the past four months, Mairéad has experienced constant diffuse pain all over her body, including greater experiences of pain, a decreased pain threshold, and increased pain ratings. Simultaneously, she has been suffering from chronic fatigue, bouts of depression, and prolonged periods of non-restorative sleep. For the pain, she has found that simple analgesics are not effective. And the pain, fatigue, and depression are all such that she has had to substantially reduce her participation in her usual sporting activities. Nevertheless, when she wakes up in the morning, it is still with the feeling in her body that she needs to go for her morning run. And, at the weekend, she feels distressed and “out of sorts” when she can’t play tennis, go cycling, or climb a mountain with her friends. These feelings manifest in her body, and she reports experiencing uncontrollable movements (clenched jaw, furrowed brow, pacing, fidgety hands, crossed arms, foot-tapping, restless legs). After several consultations with her GP, a rheumatologist, and a neurologist, Mairéad has recently been diagnosed with fibromyalgia—a common cause of chronic diffuse pain, for which there is currently no cure. Mairéad’s GP informs her about the implications of her condition and her treatment options, which include different sets of medications and psychological therapies. The GP makes the point that no matter which set of treatments Mairéad chooses, she will need to make lifestyle changes*, *specifically, refraining from the types of physical exertion required, and physical joy created by her usual recreational activities. Mairéad is extremely upset. She later describes her experience as “having the whole world pulled from under me.” She informs the GP that she “can’t decide.” The GP says that it is important to start treatment as soon as possible. Mairéad says that needs to “think about it.”*

One might query the autonomy basis for Mairéad’s inability to decide upon a specific set of treatments. Again, if we go by conventional approaches and conceive autonomy purely in terms of cognitive capacities and cognitive processes, we might infer that Mairéad is, at that moment, unable to understand, retain, or epistemically use the pertinent medical information provided by the GP. In other words, like with the previous case, purely cognitive approaches to autonomy would lead us to look for the source of the inhibition at the level of the patient’s competence and exercise of their epistemic abilities. However, the concept of embodied autonomy gestures at a more fundamental reason for Mairéad’s response and her inability to choose at that moment in time in a way that one would reasonably consider to be a genuine exercise of her autonomy.

A substantive part of Mairéad’s practical identity is bound up with her recreational activities, which she describes as her “way of life.” She expresses her practical identity and achieves a state of bodily equilibrium or poise in her environment though these routine activities. As a result, Mairéad’s inability to partake in her usual bodily practices at the level she did prior to the onset of her symptoms leads her to experience a general sense of bodily disunity. Because of her practical identity, her body is still disposed towards her usual recreational activities. At the same time, because of the pain, fatigue, and depression, her body is unable to act on those dispositions. Concurrently, she experiences uncontrollable movements, including clenched jaw, furrowed brow, pacing, fidgety hands, crossed arms, foot-tapping, and restless legs. Furthermore, her experience of bodily disunity is compounded when she receives the news from her GP that she is no longer able to partake in sporting activities.

As explained in section one, as a matter of phenomenological principle, an individual’s practical identity provides them with reasons for acting (Crowell 2013, 243), and expressions of that practical identity though intentional, skilful, fluid bodily actions are necessary to meaningfully access and enact these reasons so that the individual can reflectively and rationally respond to them. In Mairéad’s case, her usual practical identity and its associated values, desires, and motivations are no longer reasons that can rationally be employed to make treatment decisions. After all, there is no cure for fibromyalgia, so Mairéad is unable to act on her bodily disposition to choose a treatment that will re-establish her practical identity and thereby the bodily unity she once had. Furthermore, were Mairéad to respond to her values and desires as an amateur athlete and reject the GP’s advice and treatment recommendations in order to continue as best she can with her recreational activities, she would not be able to embody the same practical identity or express it in a way that she would find normatively satisfying and valuable precisely because the symptoms of pain, fatigue, and depression would stop her from experiencing bodily unity. According to phenomenological principles bound up with the concepts of practical identity and absorbed coping, Mairéad will continue to experience bodily disunity until such time as she embodies a new “way of life” and assume an updated practical identity with its own unique reasons for acting (e.g., by taking up new practices and/or prioritising practices associated other aspects of her practical identity, such as those associated with her being a mechanical engineer, an extrovert, and so on). This creates a problem where the exercise of autonomy in medical decision making is concerned. If Mairéad had, there and then, chosen one of the sets of therapies recommended to her by the GP, then, given that a substantial part of her practical identity is bound up with her “way of life” as an amateur athlete and there was no option for pursuing a treatment that would re-establish that practical identity, the authenticity of that choice would be cast into doubt. Of course, Mairéad might have decided on a set of therapies that best allowed her to live a way of life as a mechanical engineer, extrovert, and so on, that is, those other descriptions that, beyond being an athlete, make up her practical identity. Consequently, it is not the case that such a treatment decision would have been necessarily inauthentic and thereby non-autonomous. Nevertheless, despite possessing the necessary cognitive capacities for autonomy, Mairéad is unable to make the treatment decision. The lesson the phenomenological analysis teaches us is that the source of her autonomy inhibition can be explained in bodily terms, specifically, in terms of the effect of fibromyalgia on her practical identity, the impossibility of curing her condition and re-establishing her practical identity, and the effects of her practical identity crisis on her reasons for acting and underlying motivational structure.

Given that authenticity is a necessary criterion of the reflective process that contributes to the exercise of autonomy and given that such a reflective process presupposes pre-reflective access and engagement with one’s authentic values through bodily actions associated with one’s practical identity, Mairéad would need to redefine her practical identity in order to make a genuinely authentic and autonomous treatment decision. As Diana Meyers claims, because “we define ourselves as we act…we cannot redefine ourselves without altering our patterns of action” (Meyers 2005, 46). This process of redefinition could, in principle, consist of inhabiting, experiencing, or imaginatively projecting oneself into an updated practical identity with its own bodily practices and engagements.

## Conclusion

We have argued that, in principle, the body is a necessary component of the capacity for autonomy. Through the presentation of two pseudo-anonymised, part-fictional cases, we have also highlighted some of the implications of a phenomenological conception of embodied experience for our understanding of patient autonomy. As we have made clear, the body’s role in autonomous treatment choices will depend on a number of factors, including the patient’s condition, their characteristics, and features of the clinical encounter. We have argued that when a patient experiences bodily disunity and expresses themselves in ways which a clinician judges to be indicative of irrationality and/or a lack of autonomy, or when a patient will never again be able to embody the practical identity they had before the onset of the present condition, the autonomy of their treatment choice can substantively depend on their bodily capacities and expressions and the ways these relate to the patient’s mental capacity and cognitive processes. The phenomenological principles employed as the basis of an embodied approach to autonomy entail that physicians and medical staff should be disposed to be attentive to their patient’s state of bodily (dis)unity, on the basis of which they are able to express their practical identity and access their authentic values, desires, and motivations that inform treatment decisions. Without consideration of these principles, we are unable to adequately explain why a patient is unable to exercise their autonomy or develop theoretically robust healthcare mechanisms to support patient autonomy in these instances.

## Footnotes

1 Meyers (2005) has explicitly acknowledged some of the ways in which one’s body contributes to one’s autonomy, but, although we are, in part, motivated by her claims, her work in this area remains undertheorized. Others have considered the relationship between autonomy and the body from a phenomenological perspective (see, e.g., Mackenzie 2008a; Käll and Zeiler 2014; Hendl 2016>; Slatman et al. 2016; Lewis and Holm 2022), and we explain below how the discussions in this article relate to some of these accounts and what our contribution to this body of research is.

2 The only notable exception concerns the work of Kong (2017), who has demonstrated the importance of embodiment for mental capacity assessments involving cognitively-impaired patients.

3 We are immensely grateful for an anonymous reviewer’s request to emphasise the link between bodily intentionality and exercises of autonomy through rational reflection on one’s values.
